# Outcomes of endoscopic and microscopic transsphenoidal pituitary surgery: evidence from a systematic review, meta-analysis, and institutional experience

**DOI:** 10.1007/s10143-026-04404-9

**Published:** 2026-07-17

**Authors:** Alexandru Guranda, Dirk Lindner, Vincent Wähner, Felix Arlt, Martin Vychopen, Erdem Güresir, Johannes Wach

**Affiliations:** 1 Department of Neurosurgery, Leipzig University, University Hospital Leipzig, Liebigstraße 20, Leipzig, 04103 Germany; 2 Comprehensive Cancer Center Central Germany, Partner Site Leipzig, Leipzig, 04103 Germany

**Keywords:** Transsphenoidal pituitary surgery, Endoscopic approach, Microscopic approach, Gross total resection, Meta-analysis

## Abstract

To compare surgical outcomes between endoscopic and microscopic transsphenoidal pituitary surgery using international comparative evidence and an institutional cohort. A PRISMA-based systematic review and meta-analysis of comparative transsphenoidal pituitary surgery studies was performed. Gross total resection was the primary endpoint. Secondary outcomes included operative time, intraoperative blood loss, cerebrospinal fluid leak, epistaxis, meningitis, diabetes insipidus, SIADH, and visual outcomes. Fixed- and random-effects models were applied. Fifty-six comparative studies including 26,295 patients from 23 countries were analyzed (13,575 endoscopic, 12,534 microscopic), supplemented by an institutional cohort of 142 patients. Endoscopic surgery was associated with higher gross total resection rates (random-effects RR 1.08, 95% CI 1.02–1.15). Operative time did not differ significantly between techniques (random-effects MD + 3.57 min, 95% CI − 17.25 to 24.40). Intraoperative blood loss was higher in endoscopic surgery (MD 44.0 mL, 95% CI 0.94–87.08). Rates of cerebrospinal fluid leak, epistaxis, meningitis, transient and permanent diabetes insipidus, SIADH, and postoperative visual deterioration or improvement were comparable between approaches. Both techniques demonstrate comparable perioperative safety, while endoscopic surgery seems to be associated with higher resection rates overall.

**Graphical abstract:**

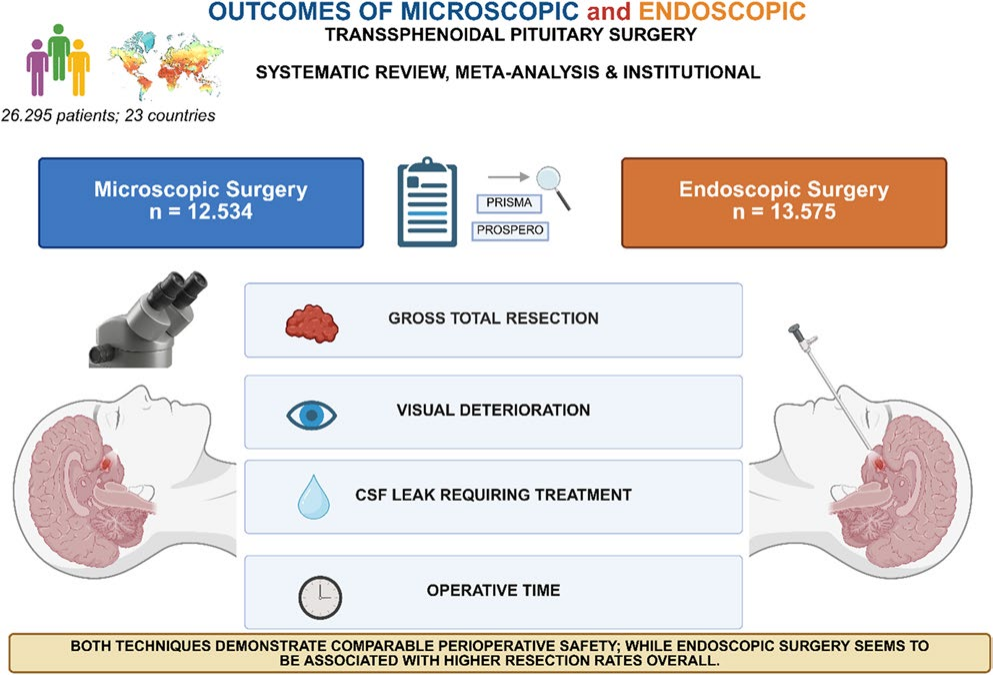

**Supplementary Information:**

The online version contains supplementary material available at 10.1007/s10143-026-04404-9.

## Introduction

Transsphenoidal surgery (TSS) is the established operative approach for pituitary adenomas (PA) and other sellar lesions [1]. Continuous advances in microsurgical instrumentation, endoscopic technology, imaging, anesthesia, and perioperative management have substantially improved surgical safety as well as endocrine and visual outcomes. Despite these developments, the optimal surgical approach—microscopic or endoscopic—remains a matter of ongoing debate.

Microscopic transsphenoidal surgery (MTS) has a long-standing track record, offering predictable anatomical orientation, stable depth perception, and well standardized operative workflows. It enables precise bimanual dissection under direct binocular visualization and remains widely practiced, particularly in centers with extensive microsurgical expertise. Endoscopic transsphenoidal surgery (ETS) was introduced to overcome limitations of the microscopic line of sight, providing a wider field of view, superior illumination, and angled visualization of the sellar and parasellar compartments [2]. While these features may facilitate tumor exposure in selected cases, ETS introduces different ergonomic demands and a distinct learning curve [3].

Recent high-quality comparative studies have yielded conflicting results. The most recent randomized controlled trial reported high gross total resection (GTR) rates without significant differences between microscopic and endoscopic approaches, although the study was limited by premature termination and small sample size [4]. In contrast, a large nationwide registry study from China using propensity score matching demonstrated comparable GTR rates in the overall cohort and slightly higher GTR rates for ETS after matching, without an increase in major complications [5]. Conversely, a large international multicenter propensity score–matched analysis of 2826 patients reported higher GTR rates for MTS, albeit at the cost of increased perioperative morbidity and resource utilization, while short-term postoperative outcomes remained similar between techniques [6].

In addition, a recently published systematic review and meta-analysis reported comparable safety and efficacy between ETS and MTS; however, the analysis was limited by heterogeneous inclusion criteria and did not comprehensively assess operative time as a surgical outcome [7]. Importantly, perioperative surgical metrics, including operative time and procedure-related operative parameters – have not been systematically synthesized, despite their relevance for surgical workflow, operative efficiency, and healthcare resource utilization.

Beyond these landmark studies, numerous comparative series have evaluated ETS and MTS with respect to extent of resection (EOR), operative parameters, and perioperative morbidity, with inconsistent findings. Interpretation is complicated by heterogeneity in study design, patient selection, tumor characteristics, surgeon experience, and outcome definitions. Many reports are single-center, underpowered, or selectively report outcomes, limiting generalizability.

Therefore, a comprehensive synthesis of contemporary comparative data that incorporates both classical surgical outcomes and perioperative surgical metrics is warranted. The present study integrates international evidence with institutional experience to systematically assess surgical outcomes and perioperative morbidity of ETS versus MTS.

## Materials and methods

### Search strategy and data collection

This study was prospectively registered in the International Prospective Register of Systematic Reviews (PROSPERO; registration ID: CRD420261282633). Clinical trial number: not applicable. A systematic literature search was conducted in PubMed and Google Scholar from their inception to January 2026 to identify comparative studies on ETS and MTS in pituitary surgery. Search terms included combinations of “pituitary adenoma,” “pituitary neuroendocrine tumor,” “transsphenoidal surgery,” “endoscopic,” and “microscopic,” supplemented by relevant Medical Subject Headings (MeSH). No initial restrictions on study design were applied. Only full-text articles published in English were included. In addition, the reference lists of all eligible articles were manually screened to identify further relevant studies not captured by the electronic search. The review was conducted in accordance with the Preferred Reporting Items for Systematic Reviews and Meta-Analyses (PRISMA) guidelines (see Supplementary Material 1). The full study protocol is provided in Supplementary Material 2.

### Eligibility criteria

Studies were eligible if they met the following criteria: (1) Population: Patients undergoing TSS for PAs or other sellar lesions. (2) Intervention: Direct comparison between ETS and MTS. (3) Outcomes: Reporting at least one predefined outcome of interest, including gross total resection (GTR), operative time, intraoperative blood loss, or postoperative complications. (4) Study design: Comparative retrospective or prospective studies. Studies were excluded if they were non-English publications, lacked a clearly defined surgical approach (purely MTS or purely ETS), failed to report relevant clinical outcomes, or enrolled fewer than 20 patients per study arm.

### Institutional cohort

A retrospective institutional cohort of adult patients with PA treated by TSS between 2012 and 2024 was analyzed. Patients underwent either ETS or MTS transsphenoidal surgery. Data were retrospectively extracted from medical records using the same definitions as in the systematic review. GTR was defined as no residual tumor on postoperative magnetic resonance imaging performed at 3-month follow-up. Resections with an extent of resection ≥ 90% were considered near-total [8]. The institutional cohort was included in the pooled analyses.

### Outcome definitions and data extraction

Outcome definitions varied across the included studies and could not be fully standardized, representing a potential source of bias. Data were extracted using a standardized form capturing study characteristics (author, year, country, and study design), patient numbers, and surgical approach (ETS vs. MTS). GTR rates were extracted as given in the individual studies. Heterogeneity regarding evaluation of GTR was present (e.g., volumetrically/two-dimensional, immediately postoperative or at 3-months after surgery). Secondary endpoints included operative time, intraoperative blood loss, CSF leak requiring invasive treatment (defined as lumbar drainage and/or surgical reintervention), epistaxis, meningitis, syndrome of inappropriate antidiuretic hormone secretion (SIADH), transient and permanent diabetes insipidus, and visual outcomes. CSF leaks irrespective of treatment were additionally analyzed in supplementary analyses due to inconsistent reporting. Visual deterioration was defined as any postoperative worsening of visual acuity or visual fields compared with baseline. Operative time and blood loss were analyzed as reported, acknowledging heterogeneity in definitions.

### Quality assessment

Methodological quality and risk of bias were assessed using the National Institutes of Health (NIH) Quality Assessment Tool for observational cohort and cross-sectional studies. Evaluated domains included clarity of objectives, patient selection, group comparability, outcome measurement, adequacy of follow-up, and statistical analysis. Two reviewers (A.G. and J.W.) independently assessed each study and rated methodological quality as good, fair, or poor. Disagreements were resolved by consensus of a third reviewer (M.V.). Common limitations across studies included retrospective design, potential selection bias, and heterogeneous outcome definitions.

### Statistical analysis

Meta-analyses were performed using the R package *meta* in R version 4.5.2 (R Foundation, Vienna, Austria). Dichotomous outcomes, including GTR and postoperative complications, were pooled as risk ratios (RRs) with 95% confidence intervals (CIs), and continuous outcomes as mean differences (MDs) with 95% CIs. Reported medians were converted to means and standard deviations when required [9]. Fixed- and random-effects models were applied using the DerSimonian–Laird method. Heterogeneity was assessed with Cochran’s Q test and the I² statistic. Statistical significance was set at *p* < 0.05.

## Results

### Study selection and characteristics

**Fig. 1:**
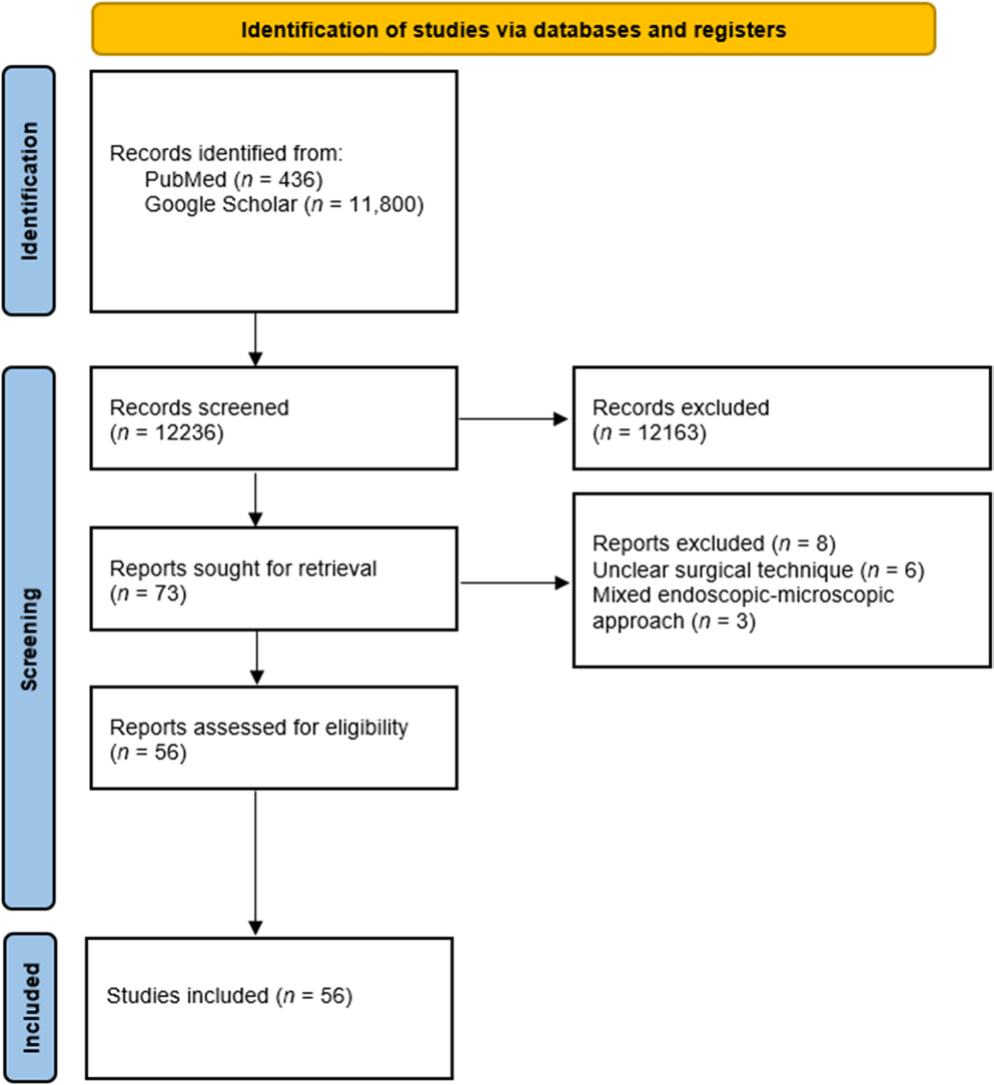
PRISMA flow diagram illustrating study identification, screening, eligibility assessment, and inclusion

The literature search identified 12,236 records comparing ETS and MTS. After screening, 73 reports were assessed in full text, of which 56 comparative studies met the inclusion criteria and were included in the final analysis (see Fig. 1). Studies were published between 2002 and 2025 and originated from 23 countries. In total, 26,295 patients were analyzed (13,575 ETS, 12,534 MTS), with additional data from an institutional cohort of 142 patients (121 ETS, 21 MTS). Three studies were based on large databases, including two studies using Truven MarketScan [10] and TriNetX [11] from the United States and one study using the National Brain Tumor Registry of China (NBTRC) [5]. Most studies were retrospective, with 42 retrospective [5, 6, 8, 10–48], 8 prospective [49–56], 5 randomized [4, 57–60], and 1 mixed-design study [61]. Mean age was reported in 30 studies and was comparable between groups (ETS: 51.2 ± 15.4 years; MTS: 51.0 ± 15.8 years). Sex distribution was available in 46 studies, including 5,958 male patients in the ETS group and 5,127 in the MTS group. Key study characteristics are summarized in Supplementary Table 1.

### Gross total resection

**Fig. 2:**
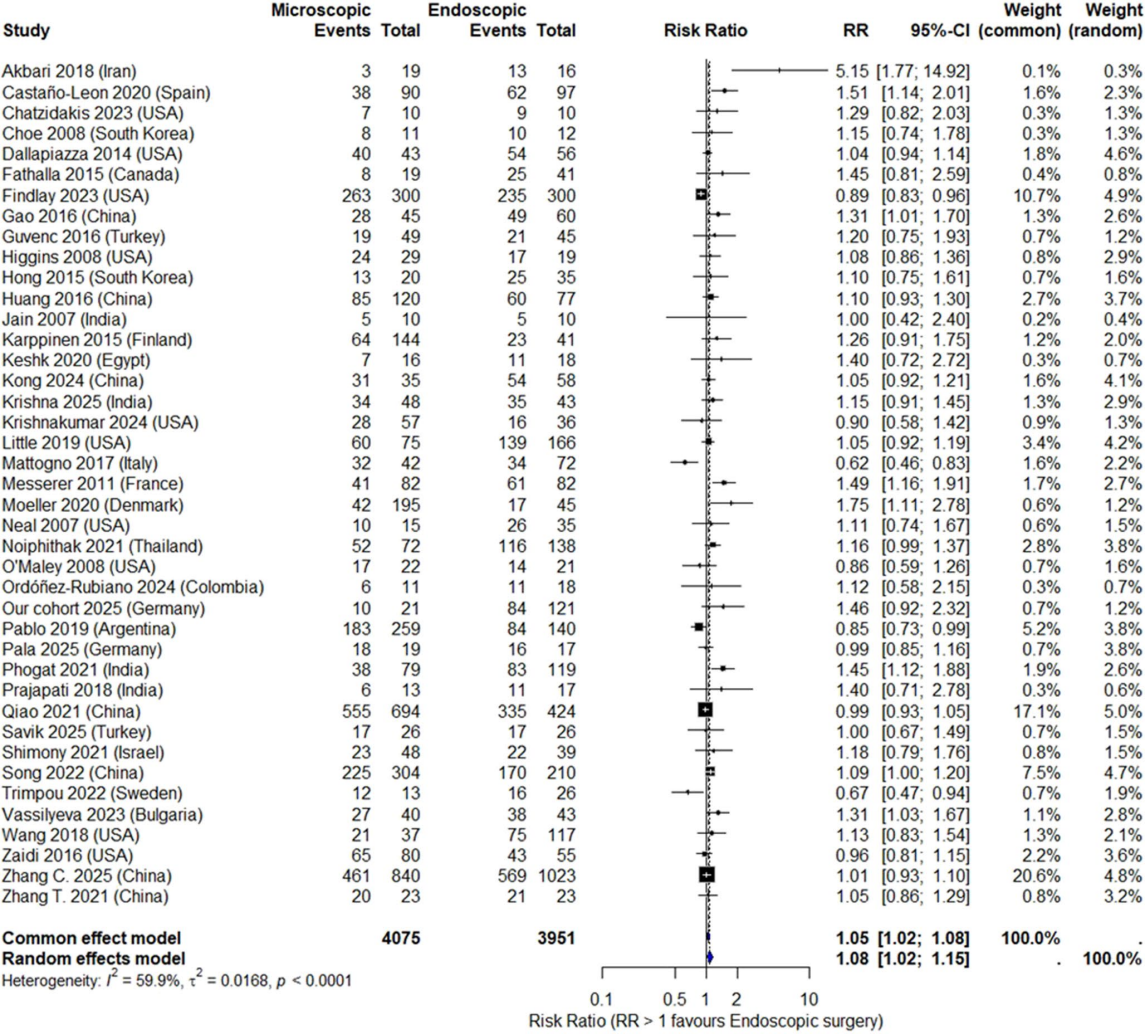
Forest plot illustrating GTR rates comparing ETS and MTS across all included comparative studies. Individual study RRs with 95% CIs are shown, together with the number of events and total patients per group. Study weights for both common- and random-effects models are displayed. The diamond at the bottom represents the pooled effect estimate for GTR across all studies

GTR was the primary endpoint of this analysis and was reported in 41 comparative studies (see Fig. 2) [4–6, 8, 13, 15, 16, 20, 21, 23, 25, 28–32, 34–40, 42, 43, 45–49, 51–54, 56, 58–61]. In the pooled analysis, ETS was associated with a higher likelihood of achieving GTR compared with MTS. The random-effects model demonstrated a statistically significant advantage for ETS (RR 1.08, 95% CI 1.02–1.15), while the common-effects model showed a smaller but consistent effect (RR 1.05, 95% CI 1.02–1.08). Moderate heterogeneity was observed across studies (I² = 59.9%, *p* < 0.0001). In the institutional cohort, GTR was achieved in 84 of 121 ETS cases (69.4%) and 10 of 21 MTS cases (47.6%). When analyzed separately using a two-sided Fisher’s exact test, this difference did not reach statistical significance (*p* = 0.078).

### Visual deterioration

**Fig. 3:**
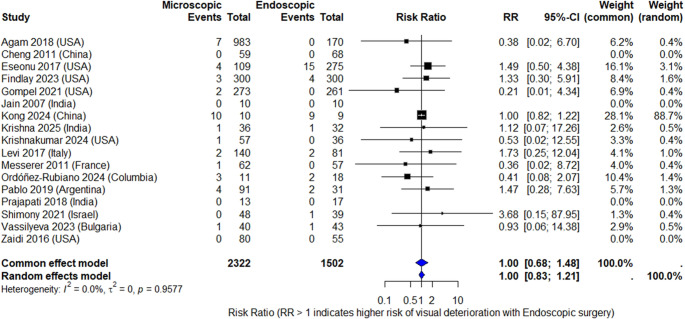
Forest plot illustrating postoperative visual deterioration comparing ETS and MTS across all included comparative studies. Individual study RRs with 95% CIs are shown, together with the number of events and total patients per group. Study weights for both common- and random-effects models are displayed. The diamond at the bottom represents the pooled effect estimate for visual deterioration across all studies

Postoperative visual deterioration was reported in 17 comparative studies [6, 8, 12, 17, 19, 22, 30–33, 35, 40, 53, 54, 56, 58, 60]. In the pooled analysis, no significant difference in the risk of visual deterioration was observed between ETS and MTS. The random-effects model showed comparable outcomes between techniques (RR 1.00, 95% CI 0.83–1.21), consistent with the common-effects model (RR 1.00, 95% CI 0.68–1.48). No statistical heterogeneity was detected (I² = 0.0%, *p* = 0.96) (see Fig. 3). The institutional cohort was not included because postoperative visual outcomes were not consistently documented.

### Cerebrospinal fluid leak requiring treatment

**Fig. 4:**
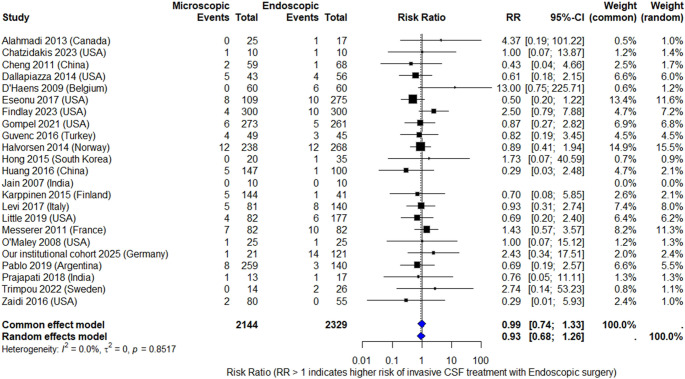
Forest plot illustrating postoperative CSF leak requiring invasive treatment comparing ETS and MTS across all included comparative studies. Individual study RRs with 95% CIs are shown, together with the number of events and total patients per group. Study weights for both common- and random-effects models are displayed. The diamond at the bottom represents the pooled effect estimate for visual deterioration across all studies

Postoperative CSF leak requiring invasive treatment was reported in 23 comparative studies (see Fig. 4) [6, 14, 16–19, 22–24, 26, 28, 29, 33, 35, 39, 40, 45, 49, 52, 54, 56, 58]. In the pooled analysis, no significant difference in the risk of CSF leak requiring treatment was observed between ETS and MTS. The random-effects model demonstrated comparable rates between techniques (RR 0.93, 95% CI 0.68–1.26), consistent with the common-effects model (RR 0.99, 95% CI 0.74–1.33). No statistical heterogeneity was detected (I² = 0.0%, *p* = 0.85).

### Operative time

**Fig. 5:**
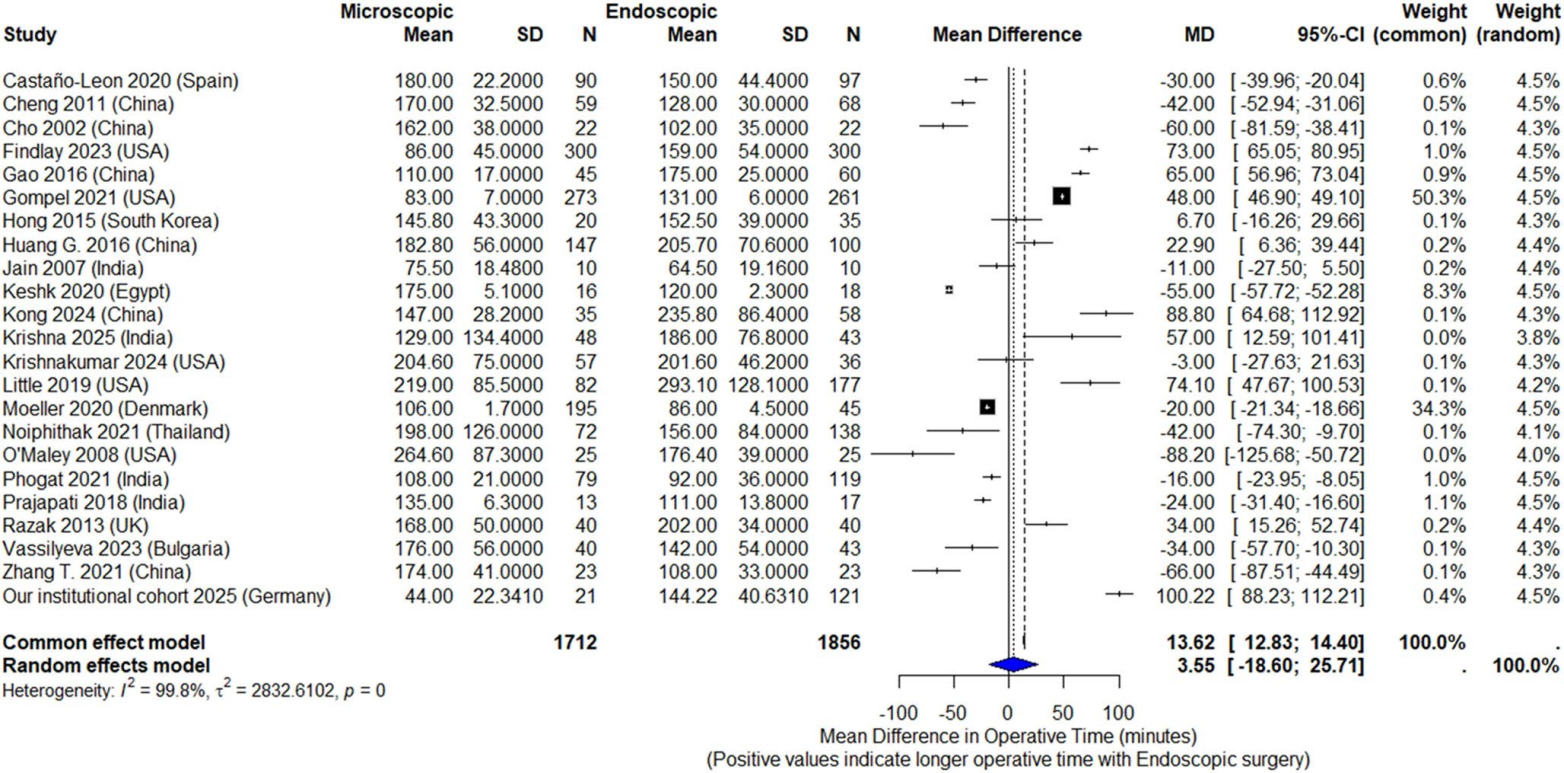
Forest plot illustrating operative time comparing ETS and MTS. Individual study MDs in minutes with 95% CIs are shown, together with study-specific means, standard deviations, and sample sizes. Study weights for both common- and random-effects models are displayed. The diamond represents the pooled effect estimate. Positive values indicate longer operative time with ETS

Operative time was reported in 23 comparative studies (see Fig. 5) [6, 15, 17, 21, 22, 26, 28, 30–32, 36, 38, 39, 47, 51, 52, 54, 55, 57, 58, 60, 61]. Based on pooled descriptive data, mean operative time was 163.88 ± 81.09 min for endoscopic transsphenoidal surgery (ETS, *n* = 1,856) and 133.18 ± 72.19 min for microscopic transsphenoidal surgery (MTS, *n* = 1,712). In the meta-analysis, no significant difference in operative time was observed between techniques. The random-effects model demonstrated a MD of + 3.57 min (95% CI − 17.25 to 24.40), whereas the common-effects model showed a statistically significant difference (MD + 13.62 min, 95% CI 12.83 to 14.40). Substantial heterogeneity was present across studies (I² = 99.8%, *p* < 0.001).

### Additional postoperative outcomes

Additional postoperative outcomes were analyzed as supplementary endpoints. Epistaxis did not differ significantly between ETS and MTS in pooled analyses (random-effects RR 1.36, 95% CI 0.99–1.87; *p* = 0.06; see Supplementary Fig. 1) [4, 6, 10, 12, 15, 17–21, 28–30, 32, 33, 35, 38, 40, 42, 44–47, 49, 52, 53, 56, 57, 60]. Transient [6, 12, 14, 15, 17, 20, 23, 28, 30–32, 38–44, 48, 49, 54, 55, 58, 60, 61] and permanent [8, 10–13, 15, 17–20, 22, 23, 25, 27–29, 31, 33, 35, 37, 39, 40, 42, 44, 49, 51, 52, 56] diabetes insipidus did not differ significantly between ETS and MTS in random-effects meta-analyses (transient DI: RR 0.94, 95% CI 0.76–1.16; *p* = 0.53; permanent DI: RR 0.89, 95% CI 0.66–1.20; *p* = 0.44; see Supplementary Figs. 2 and 3). Postoperative meningitis was reported in 31 comparative studies and did not differ significantly between ETS and MTS in either the random-effects model (RR 1.08, 95% CI 0.76–1.52; *p* = 0.56) or the common-effects model (RR 1.16, 95% CI 0.90–1.48; *p* = 0.23; see Supplementary Fig. 4) [4, 6, 10–15, 18, 19, 21, 23, 24, 28, 29, 31–33, 35–38, 40, 41, 45, 46, 48, 52, 56, 60, 61]. Postoperative CSF leak did not differ significantly between ETS and MTS in the random-effects model (RR 1.10, 95% CI 0.90–1.36; *p* = 0.36), whereas the common-effects model indicated a higher risk with ETS (RR 1.39, 95% CI 1.25–1.54; *p* < 0.001; see Supplementary Fig. 5) [4–6, 8, 10–31, 33, 35–56, 58, 61]. Intraoperative blood loss was higher in ETS compared to MTS in the random-effects model (MD 44.0 mL, 95% CI 0.94–87.08; *p* = 0.045), with a consistent effect observed in the common-effects model (MD 31.8 mL, 95% CI 27.7–35.8; *p* < 0.001), albeit in the presence of substantial heterogeneity (I² = 98.3%; see Supplementary Fig. 6) [28, 30–32, 38, 51, 54, 58, 61]. Visual improvement did not differ significantly between ETS and MTS in the random-effects model (RR 0.89, 95% CI 0.72–1.14; *p* = 0.40), despite substantial heterogeneity across studies (I² = 77.4%; see Supplementary Fig. 7) [6, 8, 15–17, 19, 21, 24, 29, 31, 32, 35, 38, 40, 42, 43, 47, 57, 60]. Postoperative SIADH did not differ significantly between ETS and MTS (random-effects RR 0.87, 95% CI 0.56–1.36; *p* = 0.55; I² = 51.7%; see Supplementary Fig. 8 [6, 10, 11, 17, 18, 28, 39, 49].

### Bias and quality evaluation

Most studies clearly defined their research question and population, but only few reported sample size calculations. Blinding was rarely performed, and adjustment for confounding factors was inconsistent. Overall methodological quality ranged from good to fair. Detailed NIH quality assessments are provided in Supplementary Table 2.

## Discussion

This systematic review and meta-analysis integrates international comparative evidence with institutional experience to evaluate surgical outcomes and perioperative morbidity of ETS versus MTS for PAs and other sellar lesions. In total, 56 comparative studies comprising 26,295 patients were included, supplemented by an institutional cohort of 142 patients.

Pooled analyses demonstrated a modest but statistically significant association between ETS and higher gross total resection rates (RR 1.08, 95% CI 1.02–1.15). However, this effect must be interpreted with caution. Individual studies showed considerable variability, with some reporting higher GTR rates following ETS [15, 47, 52, 61], while others demonstrated comparable outcomes or even favored MTS [6, 40, 43, 59]. This heterogeneity suggests that the observed meta-analytic effect does not represent a uniform technical superiority of one approach, but rather reflects the aggregation of diverse institutional practices and patient populations. Our institutional cohort demonstrated a similar numerical trend toward higher GTR rates with endoscopic surgery (69.4% vs. 47.6%), although this difference did not reach statistical significance. Furthermore, there are no standardized reporting items/classification systems for EOR in surgery of PAs such as the RANO resect classification in gliomas [62]. Hence, the literature includes data from subjective surgeon’s impressions, two-dimensional measurements, and most objectively volumetry.

A recent meta-analysis reported no significant difference in GTR between ETS and MTS [7]; however, this analysis included large NCDB cohorts in which microscopic transsphenoidal surgery was not distinguished from open craniotomies and both were classified as non-endoscopic surgery [63]. To avoid this misclassification bias, such cohorts were excluded from the present analysis. Another meta-analysis published in 2014 reported higher GTR rates in favor of ETS; however, this analysis was based on a limited number of early comparative studies and may not fully reflect contemporary surgical practice [64]. In contrast, a more recent meta-analysis published in 2022 found no significant difference in gross total resection between ETS and MTS [65].

Operative time exhibited divergent results depending on the analytical model. The mean difference was 3.57 min. While common-effects analyses suggested longer operative times for endoscopic surgery, this difference was not confirmed in random-effects analyses that accounted for substantial interstudy heterogeneity. The wide dispersion of study-level estimates, with operative times favoring either technique across individual series, indicates that operative duration is not intrinsically determined by the surgical approach itself. Rather, it appears to be shaped by surgeon experience with the technique, institutional setup, case complexity, and the stage of adoption along the learning curve. However, it has to be noted that the measurement of the operative time might be also heterogeneous (e.g. operative time, general anesthesia time). Irrespective of the surgical technique, shortened operative time is an important variable keeping the risk of postoperative infections as low as possible [66].

Direct comparisons of intraoperative complications between approaches are limited and inconsistent. In the present investigation we found no differences regarding CSF leaks. After propensity matching and multivariable adjustment, we found microscopic surgery had higher intraoperative complication odds (OR 2.22), driven mainly by about a twofold higher CSF leak risk. Some studies agree (e.g., Slot et al. meta-analysis: higher CSF leak risk with microscopy; OR 1.4) [67], while others report the opposite (e.g., Gompel et al.: endoscopic 33% vs. microscopic 10%, *p* < 0.01) [22], suggesting leaks may reflect tumor factors and surgical skills more than approach. We found no approach-related differences in major, minor, or overall postoperative complications. Tumor-related features seem to be more responsible for postoperative CSF leaks. Furthermore, sellar reconstructions techniques are highly heterogeneous [68].

In our evaluation of other adverse outcomes, we did not observe meaningful differences between approaches in postoperative complication rates. This included CSF leakage, SIADH, and other negative events like postoperative vision loss or meningitis. Our findings mirror those reported by Al-dardery et al. [7], Chen et al. [69] and Li et al. [70], and are in line with the conclusions of larger reviews by Guo et al. [71] and Chen et al. [65]. Conversely, Gao et al. [64] described comparatively lower incidences of diabetes insipidus, hypothyroidism, and septal perforation with ETS, implying a possible safety benefit, and also noted shorter admissions and fewer meningitis cases. However, we did not detect similar advantages in our dataset, which may reflect differences in regional perioperative antibiotic protocols or perioperative endocrinological therapy (e.g. hydrocortisone substitution).

The results of this study emphasize that the choice between endoscopic and microscopic transsphenoidal surgery should not be guided by an expectation of inherent superiority in safety or efficacy. Instead, optimal outcomes are likely achieved when each technique is applied within a structured environment that aligns with surgeon expertise, institutional experience, and patient-specific anatomical considerations. Centers with long-standing microsurgical expertise may achieve outcomes comparable to those reported in endoscopic series, while high-volume endoscopic centers may realize advantages within their established workflows. Novel techniques such as augmented-reality are already integrated in both ETS and MTS, which will potentially even more enhance outcomes [72, 73]. Furthermore, novel micro-inspection tools such as QEVO (Carl Zeiss Meditec, Germany) combine endoscopic view in a microsurgical setting and will have to be investigated in the future [74].

### Limitations

Several limitations warrant consideration. Most included studies were retrospective, with inherent selection bias and residual confounding; randomized and large registry studies were comparatively few. Outcome definitions and reporting (e.g., GTR, visual outcomes, CSF leak) varied substantially, potentially affecting pooled estimates. Registry-based studies, while large, depend on coding accuracy and may introduce misclassification and unmeasured confounding. Key clinical factors (tumor size/invasiveness, Knosp grade, hormonal status, surgeon experience) were inconsistently reported, limiting adjustment for case complexity and learning-curve effects. Operative time and blood loss showed marked heterogeneity across institutions and adoption eras. Finally, the accompanying institutional cohort was small and some outcomes were incompletely documented, limiting inclusion in pooled analyses.

## Conclusion

ETS and MTS demonstrate comparable perioperative safety and overall clinical outcomes. Although ETS showed a modest population-level advantage in GTR, this effect was heterogeneous and strongly influenced by institutional and surgeon-specific factors.

## Supplementary Information

Supplementary Material 1.

Supplementary Material 2.

Supplementary Material 3.

## Data Availability

The datasets generated and/or analyzed during the current study are available from the corresponding author on reasonable request.
