# Supplementary material for: Outcomes of endoscopic and microscopic transsphenoidal pituitary surgery: evidence from a systematic review, meta-analysis, and institutional experience
Source: Neurosurg Rev. 2026 Jul 17;49(1):479. doi: 10.1007/s10143-026-04404-9 (PMC13375665; doi:10.1007/s10143-026-04404-9)
Supplement: Supplementary file 1 — Supplementary Material 1. [file 10143_2026_4404_MOESM1_ESM.docx]

**Study Protocol**

**Title:**

*Comparative Outcomes of Endoscopic versus Microscopic Transsphenoidal Surgery for Pituitary Adenomas: A Systematic Review and Meta-Analysis Including an Institutional Cohort*

**Background**

Transsphenoidal surgery (TSS) is the standard surgical approach for pituitary adenomas and other sellar lesions. Both microscopic transsphenoidal surgery (MTS) and endoscopic transsphenoidal surgery (ETS) are widely used in contemporary practice. While MTS is characterized by established operative workflows and long-standing clinical experience, ETS has gained increasing adoption due to enhanced visualization of the sellar and parasellar regions.

Despite extensive clinical use of both techniques, comparative evidence regarding surgical efficacy and perioperative morbidity remains inconclusive. Randomized trials and large observational studies have reported conflicting results with respect to gross total resection, operative parameters, and postoperative complications. Interpretation of the existing literature is limited by heterogeneity in study design, patient selection, tumor characteristics, surgeon experience, and outcome definitions.

Previous systematic reviews and meta-analyses have addressed this topic (1 - 3); however, several were limited by heterogeneous inclusion criteria, incomplete assessment of operative outcomes, or exclusion of contemporary high-volume studies. Additionally, institutional outcome data are often analyzed separately and not integrated into pooled comparative analyses.

A comprehensive synthesis of current comparative evidence, combined with institutional experience using standardized outcome definitions, is therefore warranted to provide a balanced assessment of endoscopic versus microscopic transsphenoidal surgery.

**Objectives**

Primary Objective

- To compare gross total resection (GTR) rates between endoscopic and microscopic transsphenoidal surgery for pituitary adenomas.

Secondary Objectives

- To compare operative time and intraoperative blood loss between ETS and MTS.
- To assess postoperative complications, including cerebrospinal fluid (CSF) leak, epistaxis, meningitis, transient and permanent diabetes insipidus, and syndrome of inappropriate antidiuretic hormone secretion (SIADH).
- To evaluate postoperative visual outcomes, including visual improvement and deterioration.
- To integrate an institutional cohort into pooled analyses to contextualize published evidence.

**Methods**

**1. Eligibility Criteria**

- **Population:** Patients undergoing transsphenoidal surgery for pituitary adenomas or other sellar lesions.
- **Intervention:** ETS.
- **Comparator:** MTS.
- **Outcomes:** GTR, operative time, intraoperative blood loss, CSF leak (with and without invasive treatment), epistaxis, meningitis, SIADH, transient and permanent diabetes insipidus, and visual outcomes.
- **Study Design:** Comparative retrospective or prospective cohort studies.
- **Exclusion Criteria:** Non-comparative studies, studies without clear definition of surgical approach, non-English publications, studies enrolling fewer than 20 patients per treatment arm.

**2. Search Strategy**

- **Databases:** PubMed and Google Scholar.
- **Time Frame:** Database inception to January 2026.
- Search Syntax (example): PubMed: (“pituitary adenoma” OR “pituitary neuroendocrine tumor”) AND (“transsphenoidal surgery”) AND (“endoscopic” OR “microscopic”).
- **Screening Process:** Two independent reviewers screened titles and abstracts. Full texts were assessed for eligibility. Disagreements were resolved by consensus with a third reviewer.
- **Reporting Standard:** PRISMA guidelines were followed.

**3. Data Extraction**

- **Extracted Variables:** Study characteristics (author, year, country, study design), sample size per group, patient characteristics (age, sex), surgical approach (ETS vs. MTS), GTR rates, operative time, intraoperative blood loss, postoperative complications (CSF leak, epistaxis, meningitis, SIADH, diabetes insipidus), visual outcomes
- **Extraction Process:** Data were extracted using standardized collection templates. Outcome definitions were recorded as reported, acknowledging heterogeneity across studies. All extracted data were independently verified by a second investigator to ensure accuracy.

**4. Institutional Cohort**

A retrospective institutional cohort of adult patients undergoing ETS or MTS for pituitary adenomas between 2012 and 2024 was analyzed. Data were extracted from medical records using the same outcome definitions as applied in the systematic review. GTR was defined as no residual tumor on postoperative magnetic resonance imaging at 3-month follow-up. Resections with an extent of resection ≥90% were classified as near-total resection (4). The institutional cohort was included in pooled analyses.

**5. Study Quality Assessment**

Methodological quality and risk of bias were assessed using the National Institutes of Health (NIH) Quality Assessment Tool for observational cohort and cross-sectional studies. Each study was rated as good, fair, or poor based on predefined criteria, including clarity of objectives, patient selection, comparability of groups, outcome assessment, and adequacy of follow-up. Two reviewers independently performed quality assessments, with disagreements resolved by a third reviewer.

**6. Statistical Analysis**

Dichotomous outcomes were pooled as risk ratios (RRs) with 95% confidence intervals (CIs), and continuous outcomes as mean differences (MDs) with 95% CIs. When necessary, medians were converted to means and standard deviations using established methods (5). Fixed- and random-effects meta-analyses were performed using the DerSimonian–Laird method. Heterogeneity was assessed using Cochran’s Q test and the I² statistic. Statistical significance was defined as p < 0.05. Analyses were conducted using R (package meta).

**Ethics**

As this study is based exclusively on previously published data and anonymized institutional records, formal ethical approval was not required.

**Funding and Disclosures**

No external funding was received. The authors declare no conflicts of interest related to this study.

**Registration**

This study was prospectively registered in the International Prospective Register of Systematic Reviews (PROSPERO, registration ID: CRD420261282633).

**References**

1. Al-Dardery NM, Khaity A, Soliman Y, Ali MOM, Zedan EM, Muyasarah K, Elfakhrany MD. Safety and efficacy of endoscopic vs. microscopic approaches in pituitary adenoma surgery: A systematic review and meta-analysis. Neurosurg Rev. 2025 Jun 1;48(1):471. doi: 10.1007/s10143-025-03600-3. PMID: 40450149; PMCID: PMC12126332.
2. Guo S, Wang Z, Kang X, Xin W, Li X. A Meta-Analysis of Endoscopic vs. Microscopic Transsphenoidal Surgery for Non-functioning and Functioning Pituitary Adenomas: Comparisons of Efficacy and Safety. Front Neurol. 2021 Mar 23;12:614382. doi: 10.3389/fneur.2021.614382. PMID: 33833725; PMCID: PMC8021708.
3. Chen J, Liu H, Man S, Liu G, Li Q, Zuo Q, Huo L, Li W, Deng W. Endoscopic vs. Microscopic Transsphenoidal Surgery for the Treatment of Pituitary Adenoma: A Meta-Analysis. Front Surg. 2022 Feb 2;8:806855. doi: 10.3389/fsurg.2021.806855. PMID: 35187049; PMCID: PMC8847202.
4. Shimony N, Popovits N, Shofty B, Abergel A, Ram Z, Grossman R. Endoscopic transsphenoidal surgery reduces the need for re-operation compared to the microscopic approach in pituitary macroadenomas. Eur J Surg Oncol. 2021 Jun;47(6):1352-1356. doi: 10.1016/j.ejso.2021.02.004. Epub 2021 Feb 14. PMID: 33637372.
5. Wan X, Wang W, Liu J, Tong T. Estimating the sample mean and standard deviation from the sample size, median, range and/or interquartile range. BMC Med Res Methodol. 2014 Dec 19;14:135. doi: 10.1186/1471-2288-14-135. PMID: 25524443; PMCID: PMC4383202.

**Corresponding Author**
Alexandru Guranda
Department of Neurosurgery, University Hospital Leipzig
Liebigstraße 20, 04103 Leipzig, Germany
alexandru.guranda@medizin.uni-leipzig.de
