# Supplementary material for: Outcomes of endoscopic and microscopic transsphenoidal pituitary surgery: evidence from a systematic review, meta-analysis, and institutional experience
Source: Neurosurg Rev. 2026 Jul 17;49(1):479. doi: 10.1007/s10143-026-04404-9 (PMC13375665; doi:10.1007/s10143-026-04404-9)
Supplement: Supplementary file 2 — Supplementary Material 2. [file 10143_2026_4404_MOESM2_ESM.docx]

**Supplementary material 1.** PRISMA 2020 Checklist for the Systematic Review and Meta-Analysis of Endoscopic vs. Microscopic Transsphenoidal Pituitary Surgery

| Section | Item | Description | Page Number |
| --- | --- | --- | --- |
| Title | Title | Outcomes of Endoscopic and Microscopic Transsphenoidal Pituitary Surgery: Evidence from a Systematic Review, Meta-analysis, and Institutional Experience | 1 |
| Abstract | Structured Summary | A structured abstract summarizing Objective, Methods, Results, and Conclusions is provided in accordance with PRISMA recommendations. | 3 |
| Introduction | Rationale | \| The rationale for comparing endoscopic and microscopic transsphenoidal approaches is described, highlighting conflicting evidence from randomized trials, registry data, and prior meta-analyses. \| \| --- \| | 4 |
| Introduction | Objectives | The objectives are clearly stated: to compare surgical efficacy and perioperative outcomes between ETS and MTS using international comparative evidence and an institutional cohort. | 4 |
| Methods | Protocol and registration | The review protocol was prospectively registered in PROSPERO (ID: CRD420261282633) and conducted in accordance with PRISMA guidelines. | 4-5 |
| Methods | Eligibility Criteria | Inclusion and exclusion criteria are clearly defined, including population, intervention (ETS vs. MTS), outcomes (GTR, operative time, blood loss, complications), and study design. | 5 |
| Methods | Information Sources | Electronic searches were performed in PubMed and Google Scholar from inception to January 2026. Reference lists of included studies were manually screened.. | 4-5 |
| Methods | Search Strategy | Search terms included combinations of “pituitary adenoma,” “pituitary neuroendocrine tumor,” “transsphenoidal surgery,” “endoscopic,” and “microscopic.” Full search strategy is reported in the Methods section. | 4-5 |
| Methods | Selection Process | Two reviewers independently screened titles, abstracts, and full texts. Disagreements were resolved by consensus with a third reviewer. | 5-6 |
| Methods | Data Collection Process | Data extraction was performed using a standardized form capturing study characteristics, patient numbers, surgical approach, and outcomes of interest. | 5 |
| Methods | Data Items | Extracted variables included GTR, operative time, intraoperative blood loss, CSF leak, epistaxis, meningitis, diabetes insipidus, SIADH, and visual outcomes. | 5 |
| Methods | Study Risk of Bias Assessment | Methodological quality was assessed using the NIH Quality Assessment Tool for observational cohort and cross-sectional studies. | Supplementary Table 2 |
| Methods | Effect Measures | Dichotomous outcomes were pooled as risk ratios (RRs) and continuous outcomes as mean differences (MDs), both with 95% confidence intervals. | 6 |
| Methods | Synthesis of Results | Fixed- and random-effects meta-analyses were performed using the DerSimonian–Laird method. Heterogeneity was assessed using Cochran’s Q and I² statistics. | 6 |
| Methods | Risk of Bias Across Studies | Overall risk of bias and common methodological limitations across studies are summarized. | 5-6 |
| Results | Study Selection | The study selection process is presented using a PRISMA flow diagram detailing identification, screening, eligibility, and inclusion. | 6 |
| Results | Study Characteristics | Characteristics of the 56 included comparative studies (design, country, sample size, and approach) are summarized. | 6; Supplementary Table 1 |
| Results | Results of Individual Studies | Individual study results for GTR, operative time, and complications are displayed in forest plots with corresponding effect estimates. | 6-11 |
| Results | Synthesis of Results | Pooled estimates for GTR, operative time, intraoperative blood loss, CSF leak, visual outcomes, and other complications are reported using common- and random-effects models. | 6-11 |
| Results | Risk of Bias in Studies | Study-level methodological quality ratings (good/fair) are summarized and discussed. | 12 |
| Discussion | Summary of Evidence | The discussion synthesizes pooled findings, emphasizing comparable safety profiles and a modest, heterogeneous GTR advantage for ETS. | 12-13 |
| Discussion | Limitations | Limitations include retrospective study designs, heterogeneous outcome definitions, variability in surgeon experience, and limited randomized evidence. | 13 |
| Discussion | Conclusions | Conclusions highlight that ETS and MTS show comparable perioperative safety, with outcomes strongly influenced by institutional expertise rather than technique alone. | 14 |
| Funding | Funding | The study reports no external funding. | 14 |
| Acknowledgments | Contributions | Author contributions are detailed; no external writing or analytical assistance was used. | 14 |
