# Supplementary material for: Outcomes of endoscopic and microscopic transsphenoidal pituitary surgery: evidence from a systematic review, meta-analysis, and institutional experience
Source: Neurosurg Rev. 2026 Jul 17;49(1):479. doi: 10.1007/s10143-026-04404-9 (PMC13375665; doi:10.1007/s10143-026-04404-9)
Supplement: Supplementary file 3 — Supplementary Material 3. [file 10143_2026_4404_MOESM3_ESM.docx]

**Supplementary Table 1.** Study characteristics and baseline patient demographics.

| **Author** | **Country** | **Study design** | **Year** | **Cohort period** | **Age - ETS** | **Age - MTS** | **Male sex ETS (n)** | **Male sex MTS (n)** | **ETS (n)** | **MTS (n)** | **Tumor status** |
| --- | --- | --- | --- | --- | --- | --- | --- | --- | --- | --- | --- |
| Agam | USA | Retrosp. | 2018 | 1992-2017 | Mean 53.3 ±13.5 | Mean 49.1 ± 16.6 | NR | NR | 170 | 983 | Mixed |
| Akbari | Iran | Retrosp. | 2018 | 2012-2014 | Mean 39.43 ± 15.21 | Mean 43.06 ± 11.29 | 9 | 10 | 16 | 19 | Primary |
| Alahmadi | Canada | Retrosp. | 2013 | 2000-2010 | NR | NR | 3 | 8 | 17 | 25 | Primary |
| Asemota | USA | Retrosp. | 2017 | 2010-2014 | Mean 45.14 ± 13.23 | Mean 45.21 ± 13.19 | 1194 | 1453 | 2679 | 3207 | Not defined |
| Castaño-Leon | Spain | Retrosp. | 2020 | 1995-2017 | 52 (IQR = 22) | 45 (IQR =24) | 39 | 36 | 97 | 90 | Primary |
| Chatzidakis | USA | Retrosp. | 2023 | 2004-2015 | Mean 54.6 ± 12.0 | Mean 59.5 ± 13.1 | 5 | 7 | 10 | 10 | Primary |
| Cheng | China | Retrosp. | 2011 | 2003-2009 | Mean 37.2 (13-69) | Mean 33.8 (11-71) | 31 | 20 | 68 | 59 | Primary |
| Cho | China | RCT | 2002 | 1996-2000 | Mean 45.3 (22-60) | Mean 46.7 (18-56) | 0 | 1 | 22 | 22 | Primary |
| Choe | South Korea | Retrosp. | 2008 | 1997-2004 | Mean 47 ± 12 | Mean 48± 10 | 5 | 2 | 12 | 11 | Primary |
| D’Haens | Belgium | Retrosp. | 2009 | 1995-2007 | Mean 37 (10-70) | Mean 35 (10-68) | NR | NR | 60 | 60 | Primary |
| Dallapiazza | USA | Prosp. | 2014 | 2010-2013 | Mean 56.2 ± 12.8 | Mean 56.7 ± 16.9 | 27 | 24 | 56 | 43 | Primary |
| Eseonu | USA | Retrosp. | 2017 | 2005-2015 | Mean 49.0 ± 16.2 | Mean 48.8 ± 15.8 | 115 | 48 | 275 | 109 | Primary |
| Fathalla | Canada | Retrosp. | 2015 | 2000-2013 | Mean 43.2 | Mean 42.1 | 21 | 7 | 42 | 23 | Primary |
| Findlay | USA | Retrosp. | 2023 | 2017-2020 | Mean 52.8 ± 16.3 | Mean 52.6 ± 16.3 | 123 | 133 | 300 | 300 | Mixed |
| Gao | China | Retrosp. | 2016 | 2012-2014 | Mean 44.6 (19-75) | Mean 48.8 (21-77) | 26 | 19 | 60 | 45 | Primary |
| Gompel | USA | Retrosp. | 2021 | 2014-2019 | Mean 51.7 ± 0.9 | Mean 50.3 ± 0.9 | NR | NR | 261 | 273 | Mixed |
| Guvenc | Turkey | Retrosp. | 2016 | 2000-2014 | Mean 48.28 ± 14.1 | Mean 36.95 ± 12.96 | 19 | 18 | 45 | 49 | Primary |
| Halvorsen | Norway | Retrosp. | 2014 | 2002-2011 | NR | NR | NR | NR | 268 | 238 | Mixed |
| Higgins | USA | Retrosp. | 2008 | 2002-2008 | 54.2 | 52.8 | 11 | 14 | 19 | 29 | Mixed |
| Hong | South Korea | Retrosp. | 2015 | 2012-2013 | Mean 50.8 ± 13.8 | Mean 58.5 ± 12.9 | 16 | 9 | 35 | 20 | Primary |
| Huang G. | China | Retrosp. | 2016 | 2007-2014 | Mean 43.4 ± 14.0 | Mean 40.4 ± 14.2 | 59 | 53 | 100 | 147 | Primary |
| Huang Y. | China | Retrosp. | 2023 | 2019-2021 | NR | NR | 30 | 20 | 73 | 54 | Primary |
| Jain | India | RCT | 2007 | NR | Mean 40.10 | Mean 31.60 | NR | NR | 10 | 10 | Primary |
| Kahilogullari | Turkey | Prosp. | 2013 | 2010-2012 | Mean 40.84 ± 12.56 | Mean 46.56 ± 7.75 | 4 | 6 | 25 | 25 | Primary |
| Karpinnen | Finland | Retrosp. | 2015 | 2000-2010 | Mean 58.5 ± 16 | Mean 58.4 ± 13 | 23 | 95 | 41 | 144 | Primary |
| Keshk | Egypt | Prosp. | 2020 | 2015-2019 | Mean 46.3 ± 10.45 | Mean 42.5 ± 12.1 | 7 | 9 | 18 | 16 | Recurrent |
| Kong | China | Retrosp. | 2024 | 2019-2022 | Mean 50.84 ± 12.65 | Mean 49.97 ± 12.00 | 16 | 16 | 58 | 35 | Primary |
| Krishna | India | Retrosp. | 2025 | 2019-2023 | Mean 48.67 ± 16.20 | Mean 44.32 ± 12.36 | 27 | 25 | 43 | 48 | Primary |
| Krishnakumar | USA | Retrosp. | 2024 | 2009-2021 | NR | NR | 20 | 32 | 36 | 57 | Primary |
| Levi | Italy | Retrosp. | 2017 | 2004-2012 | Median 58.5 | Median 52 | 86 | 44 | 140 | 81 | Primary |
| Little | USA | Prosp. | 2019 | 2015-2017 | Mean 58.1 ± 14.0 | Mean 58.6 ± 13.3 | 104 | 52 | 177 | 82 | Mixed |
| Mattogno | Italy | Retrosp. | 2017 | 2007-2012 | Mean 51.70 (21-78) | Mean 48.35 (15-83) | 36 | 19 | 72 | 42 | Primary |
| Messerer | France | Retrosp. | 2011 | 2006-2009 | Median 57 | Median 56.50 | 47 | 51 | 82 | 82 | Primary |
| [Mø](https://pubmed.ncbi.nlm.nih.gov/?term=%22M%C3%B8ller%20MW%22%5BAuthor%5D)ller | Denmark | Retrosp. | 2020 | 2016-2017 | Median 61 | Median 58 | 25 | 107 | 45 | 195 | Mixed |
| Naimi | USA | Retrosp. | 2023 | 2010-2021 | Mean 58 ± 16.9 | Mean 58.6 ± 17.2 | 2680 | 1555 | 5470 | 3174 | Not defined |
| Neal | USA | Retrosp. | 2007 | 1990-2004 | NR | NR | NR | NR | 35 | 15 | Primary |
| Noiphithak | Thailand | Retrosp. | 2021 | 2010-2019 | Mean 48.8 ±13.9 | Mean 53.8 ± 15.6 | 77 | 44 | 138 | 72 | Mixed |
| O’Maley | USA | Retrosp. | 2008 | 2003-2008 | Mean 47.9 (18-73) | Mean 50.8 (23-78) | 15 | 16 | 25 | 25 | Primary |
| Ordóñez-Rubiano | Colombia | Prosp. | 2024 | 2018-2019 | Mean 50.2 ± 10.1 | Mean 52.3 ± 15.1 | 9 | 3 | 18 | 11 | Primary |
| Pablo | Argentina | Retrosp. | 2019 | 2006-2014 | Average 51 (17-90) | Average 48.5 (18-85) | 61 | 109 | 140 | 259 | Primary |
| Pala | Germany | RCT | 2025 | 2018-2022 | Mean 56.2 ± 14.7 | Mean 59.8 ± 15.3 | 7 | 4 | 17 | 19 | Primary |
| Phogat | India | Mix | 2021 | 2013-2018 | NR | NR | NR | NR | 119 | 79 | Primary |
| Prajapati | India | Prosp. | 2018 | NR | Mean 41.06 ± 11.75 | Mean 41.91 ± 13.23 | NR | NR | 17 | 13 | Primary |
| Qiao | China | RCT | 2021 | 2010-2019 | Mean 44.3 ± 12.7 | Mean 44.4 ± 11.8 | 185 | 334 | 424 | 694 | Mixed |
| Razak | United Kingdom | Prosp. | 2013 | 2008-2009 | Mean 47.4 (9-75) | Mean 49.3 (23-73) | 19 | 22 | 40 | 40 | Primary |
| Sarkar | India | Retrosp. | 2014 | 2005-2013 | Mean 37.6 ± 10.8 | Mean 38.7 ± 12.2 | 30 | 26 | 66 | 47 | Primary |
| Savik | Turkey | Retrosp. | 2025 | 2012-2018 | Mean 50.6 | Mean 50.2 | NR | NR | 26 | 26 | Primary |
| Shimony | Israel | Retrosp. | 2021 | 2006-2017 | Mean 57.79 ± 12.91 | Mean 52.75 ± 13.93 | NR | NR | 39 | 48 | Recurrent |
| Song | China | Retrosp. | 2022 | 2015-2020 | Mean 54 ± 12 | Mean 55 ± 12 | 100 | 147 | 210 | 304 | Primary |
| Starke | USA | Retrosp. | 2013 | 2004-2009 | Mean 49.2 ± 14.9 | Mean 47.5 ± 14.2 | 40 | 20 | 72 | 41 | Mixed |
| Trimpou | Sweden | Retrosp. | 2022 | 2003-2018 | Mean 48 | Mean 44 | 8 | 2 | 26 | 14 | Primary |
| Vassilyeva | Bulgaria | RCT | 2023 | 2017-2021 | Mean 43.26 ± 8.64 | Mean 44.12 ± 9.14 | 17 | 16 | 43 | 40 | Primary |
| Wang | USA | Retrosp. | 2018 | 2003-2012 | Mean 50 | Mean 52 | 54 | 10 | 117 | 37 | Primary |
| Zaidi | USA | Prosp. | 2016 | 2011-2014 | Mean 55.9 ± 13.8 | Mean 59.1 ± 14.6 | 35 | 50 | 55 | 80 | Mixed |
| Zhang C. | China | Retrosp. | 2025 | 2011-2021 | Mean 48 (37, 57) | Mean 47 (38, 58) | 480 | 419 | 1023 | 840 | Not defined |
| Zhang T. | China | Retrosp. | 2021 | 2017-2020 | 26-58(55.6) | 23-60 (53.2) | 13 | 12 | 23 | 23 | Primary |

**Abbreviations:** Retrosp., retrospective; Prosp., prospective; RCT, randomized controlled trial; ETS, endoscopic transsphenoidal surgery; MTS, microscopic transsphenoidal surgery; NR, not reported.


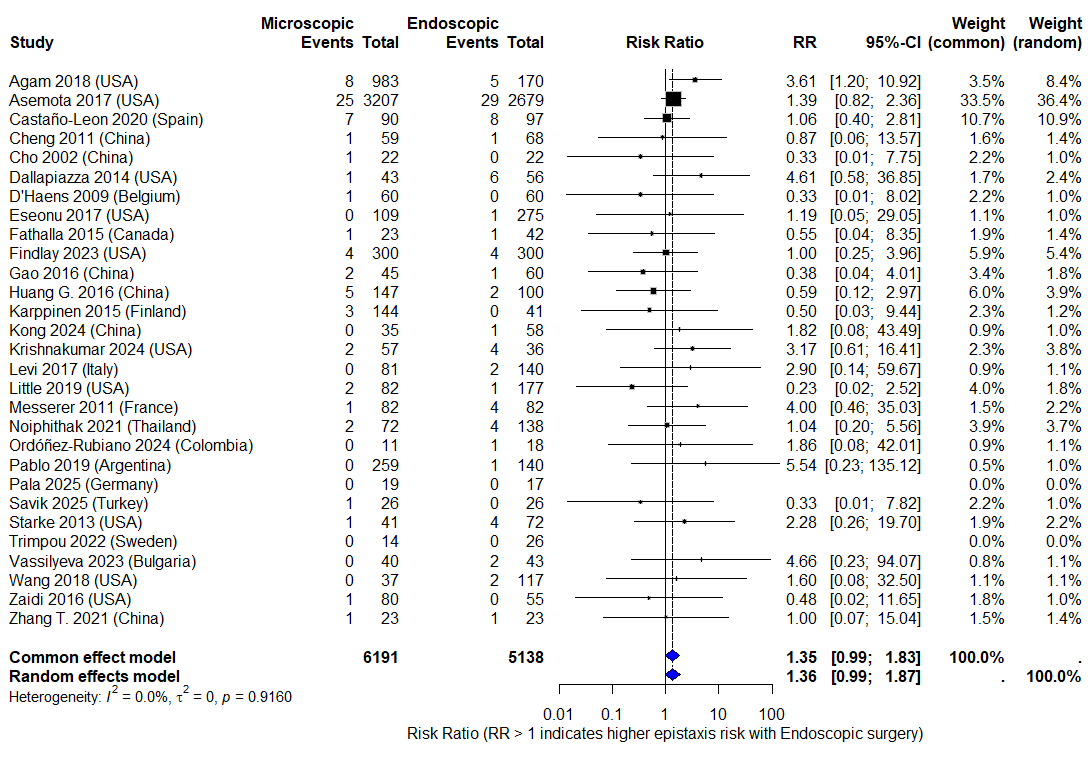


**Supplementary Figure 1.** Forest plot illustrating postoperative epistaxis comparing ETS and MTS. Individual study RRs with 95% CIs are shown, together with the number of events and total patients in each treatment arm. Study weights for both common- and random-effects models are displayed. The diamond represents the pooled effect estimate. Values greater than 1 indicate a higher risk of epistaxis with ETS.


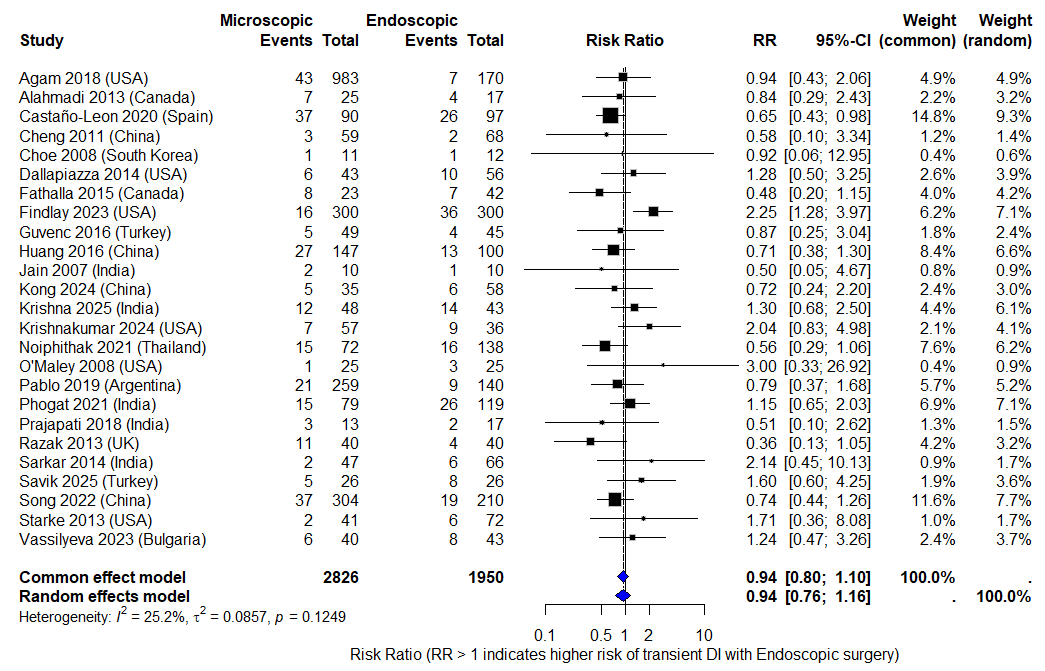


**Supplementary Figure 2.** Forest plot illustrating the risk of transient diabetes insipidus comparing ETS and MTS. Individual study RRs with 95% CIs are shown, together with the number of events and total patients in each treatment arm. Study weights for both common- and random-effects models are displayed. The diamond represents the pooled effect estimate. Values greater than 1 indicate a higher risk of transient diabetes insipidus with ETS.


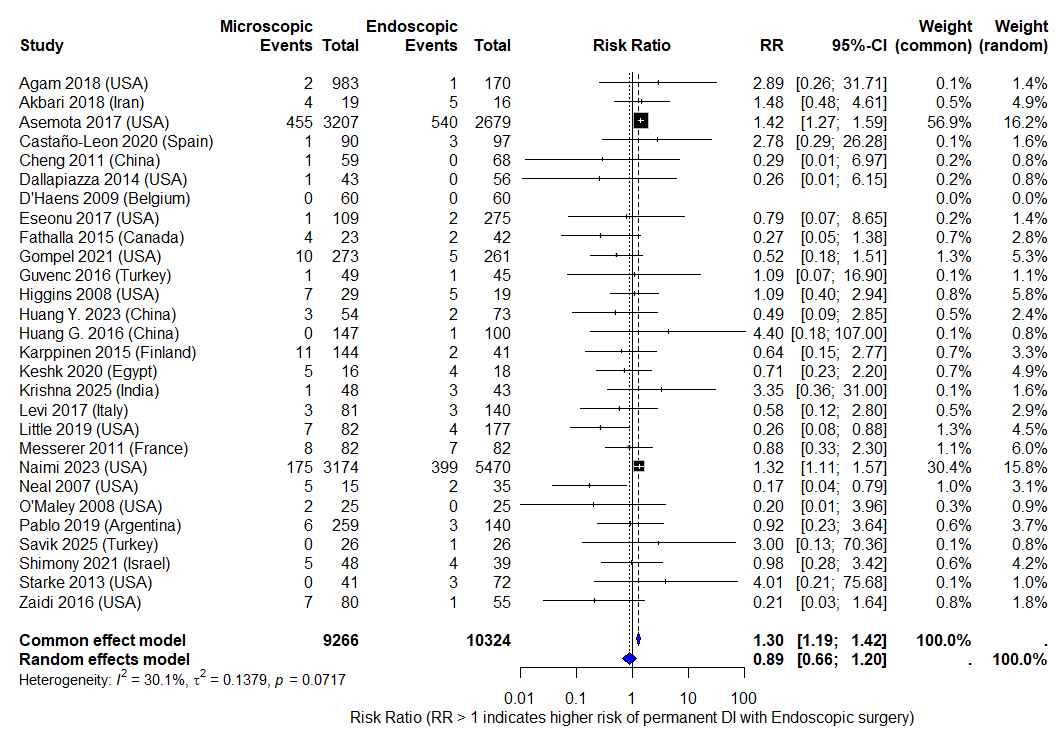


**Supplementary Figure 3.** Forest plot illustrating the risk of permanent diabetes insipidus comparing ETS and MTS. Individual study RRs with 95% CIs are shown, together with the number of events and total patients in each treatment arm. Study weights for both common- and random-effects models are displayed. The diamond represents the pooled effect estimate. Values greater than 1 indicate a higher risk of permanent diabetes insipidus with ETS.


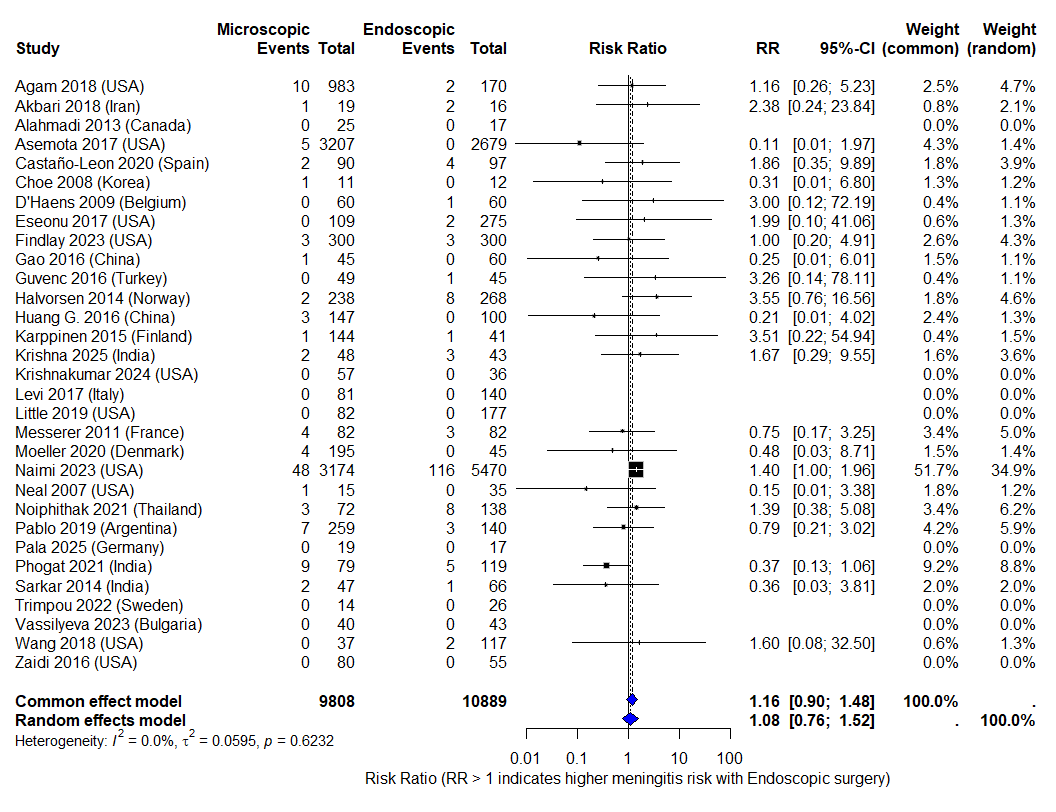


**Supplementary Figure 4.** Forest plot illustrating the risk of postoperative meningitis comparing ETS and MTS. Individual study RRs with 95% CIs are shown, together with the number of events and total patients in each treatment arm. Study weights for both common- and random-effects models are displayed. The diamond represents the pooled effect estimate. Values greater than 1 indicate a higher risk of permanent diabetes insipidus with ETS.


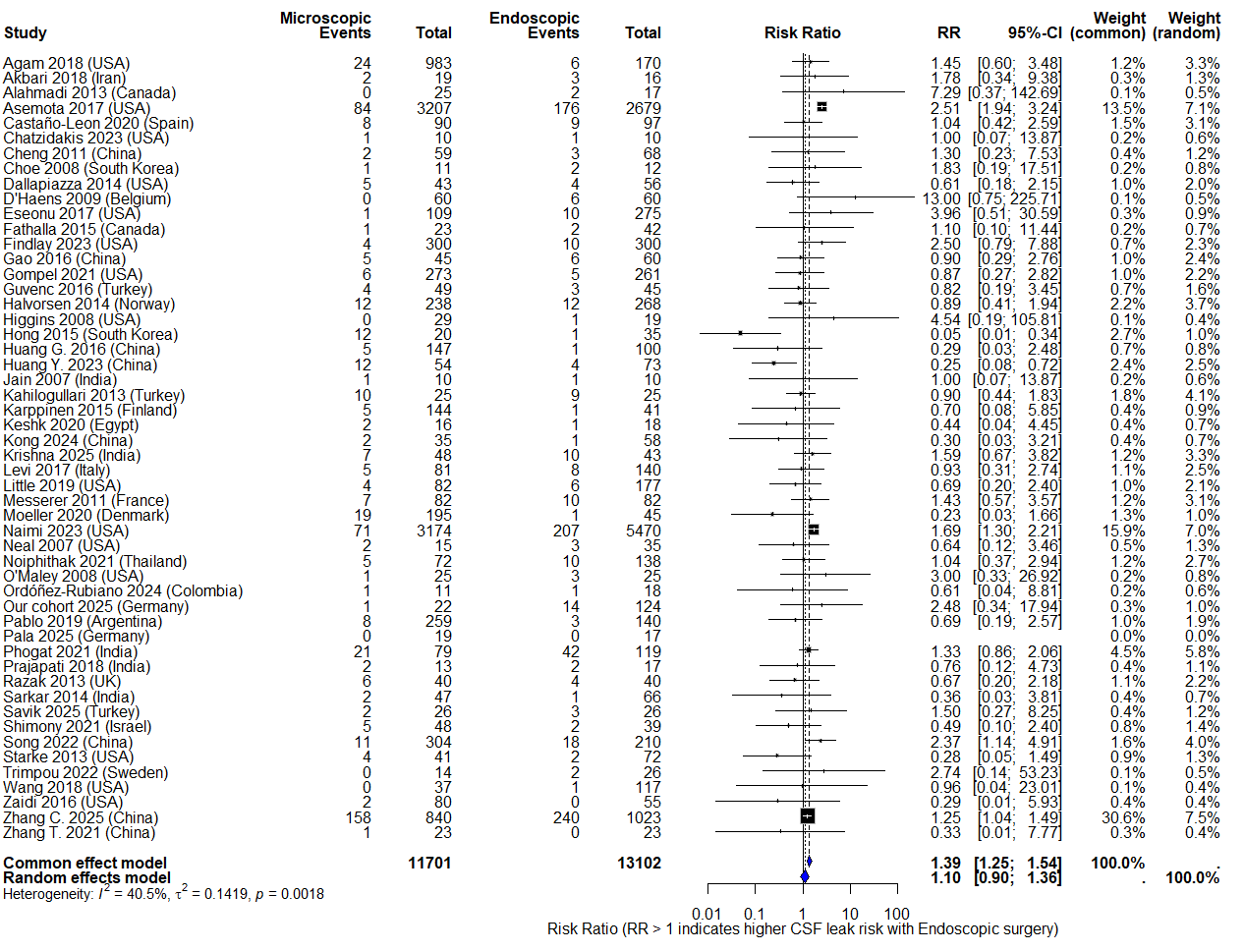


**Supplementary Figure 5.** Forest plot illustrating the risk of postoperative CSF leak comparing ETS and MTS. Individual study RRs with 95% CIs are shown, together with the number of events and total patients in each treatment arm. Study weights for both common- and random-effects models are displayed. The diamond represents the pooled effect estimate. Values greater than 1 indicate a higher risk of permanent diabetes insipidus with ETS.


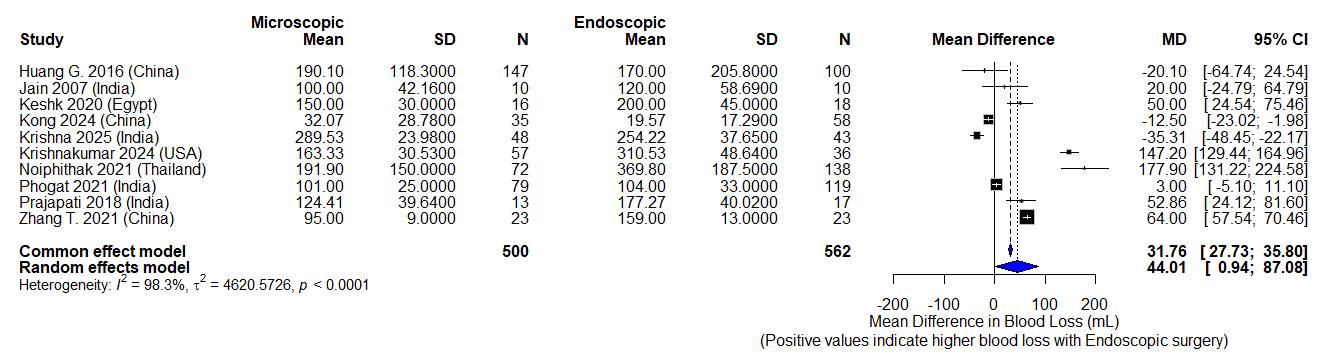


**Supplementary Figure 6.** Forest plot illustrating blood loss comparing ETS and MTS. Individual study MDs in minutes with 95% CIs are shown, together with study-specific means, standard deviations, and sample sizes. Study weights for both common- and random-effects models are displayed. The diamond represents the pooled effect estimate. Positive values indicate longer operative time with ETS.


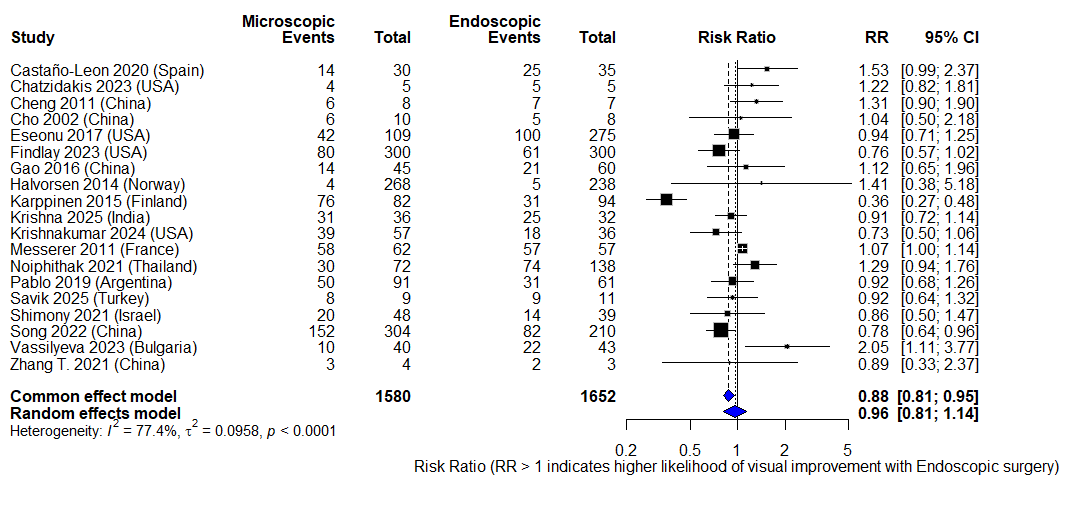


**Supplementary Figure 7.** Forest plot illustrating postoperative visual improvement comparing ETS and MTS. Individual study RRs with 95% CIs are shown, together with the number of events and total patients in each treatment arm. Study weights for both common- and random-effects models are displayed. The diamond represents the pooled effect estimate. Values greater than 1 indicate a higher risk of permanent diabetes insipidus with ETS.


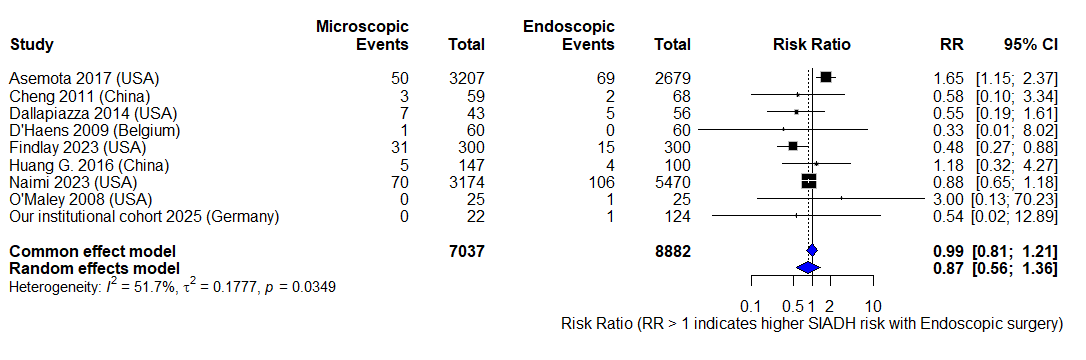


**Supplementary Figure 8.** Forest plot illustrating postoperative risk of SIADH comparing ETS and MTS. Individual study RRs with 95% CIs are shown, together with the number of events and total patients in each treatment arm. Study weights for both common- and random-effects models are displayed. The diamond represents the pooled effect estimate. Values greater than 1 indicate a higher risk of permanent diabetes insipidus with ETS.

**Supplementary Table 2. NIH Quality Assessment of Observational Studies.** This table summarizes the methodological quality assessment of included retrospective and prospective observational studies using the National Institutes of Health (NIH) Quality Assessment Tool. Each study was evaluated across predefined methodological domains and categorized as “Yes” (criterion met), “No” (criterion not met), or “Not reported (NR)” (insufficient or absent information in the original publication). The table provides an overview of methodological strengths, limitations, and reporting completeness across the included studies.

| **NIH Criteria** | **Agam MS et al.** | **Akbari H. et al.** | **Alahmadi H. et al.** | **Asemota AO et al.** | **Castaño-León AM et al.** | **Chatzidakis S. et al.** | **Cheng R-X et al.** | **Choe J-H et al.** | **D’Haens J. et al.** | **Dallapiazza R. et al.** | **Eseonu CI et al.** | **Fathalla H. et al.** | **Findlay MC et al.** | **Gao Y. et al.** | **van Gompel JJ et al.** | **Guvenc G. et al.** | **Halvorsen H. et al.** | **Higgins TS et al.** | **Hong SD et al.** | **Huang G-D et al.** | **Huang Y. et al.** | **Kahilogullari G. et al.** | **Karppinen A. et al.** | **Keshk M. et al.** | **Kong F et al.** |
| --- | --- | --- | --- | --- | --- | --- | --- | --- | --- | --- | --- | --- | --- | --- | --- | --- | --- | --- | --- | --- | --- | --- | --- | --- | --- |
| Clearly stated research question | Yes | Yes | Yes | Yes | Yes | Yes | Yes | Yes | Yes | Yes | Yes | Yes | Yes | Yes | Yes | Yes | Yes | Yes | Yes | Yes | Yes | Yes | Yes | Yes | Yes |
| Defined study population | Yes | Yes | Yes | Yes | Yes | Yes | Yes | Yes | Yes | Yes | Yes | Yes | Yes | Yes | Yes | Yes | Yes | Yes | Yes | Yes | Yes | Yes | Yes | Yes | Yes |
| ≥50% participation rate | Yes | Yes | NR | Yes | Yes | NR | Yes | NR | Yes | NR | Yes | NR | Yes | NR | Yes | NR | Yes | Yes | Yes | Yes | Yes | Yes | NR | NR | Yes |
| Uniform inclusion/exclusion criteria | Yes | Yes | Yes | Yes | Yes | NR | NR | Yes | Yes | Yes | Yes | Yes | Yes | Yes | Yes | NR | Yes | NR | Yes | Yes | Yes | Yes | Yes | Yes | Yes |
| Justification of sample size | No | No | No | No | No | No | No | No | No | No | No | No | No | No | No | No | No | No | No | No | No | No | No | No | No |
| Exposure assessed before outcome | Yes | Yes | Yes | Yes | Yes | Yes | Yes | Yes | Yes | Yes | Yes | Yes | Yes | Yes | Yes | Yes | Yes | Yes | Yes | Yes | Yes | Yes | Yes | Yes | Yes |
| Sufficient follow-up period | NR | Yes | Yes | NR | Yes | Yes | Yes | Yes | Yes | Yes | Yes | Yes | Yes | Yes | Yes | Yes | Yes | Yes | Yes | Yes | NR | Yes | Yes | Yes | Yes |
| Measurable/consistent exposure assessment | Yes | Yes | Yes | Yes | Yes | Yes | Yes | Yes | Yes | Yes | Yes | Yes | Yes | Yes | Yes | Yes | Yes | Yes | Yes | Yes | Yes | Yes | Yes | Yes | Yes |
| Clearly defined outcome measures | Yes | Yes | Yes | Yes | Yes | Yes | Yes | Yes | Yes | Yes | Yes | Yes | Yes | Yes | Yes | Yes | Yes | Yes | Yes | Yes | Yes | Yes | Yes | Yes | Yes |
| Blinded outcome assessment | No | No | No | No | No | No | No | No | No | No | Yes | No | No | No | Yes | No | No | No | No | No | No | No | No | No | No |
| ≤20% loss to follow-up | NR | NR | NR | NR | NR | NR | NR | NR | NR | NR | NR | NR | NR | NR | NR | NR | Yes | NR | NR | Yes | NR | Yes | Yes | NR | Yes |
| Confounders measured and adjusted | Yes | Yes | Yes | Yes | Yes | Yes | Yes | Yes | Yes | Yes | Yes | Yes | Yes | Yes | Yes | Yes | Yes | Yes | Yes | Yes | Yes | Yes | Yes | Yes | Yes |
| **NIH Criteria** | **Krishnakumar A. et al.** | **Levi V. et al.** | **Little AS et al.** | **Mattogno P.P. et al.** | **Messerer M. et al.** | **Møller M.W. et al.** | **Naimi B. et al.** | **Neal JG et al.** | **Noiphithak R. et al.** | **O’Malley B.W. Jr. et al.** | **Ordóñez-Rubiano E.G. et al.** | **Pablo et al.** | **Phogat V. et al.** | **Prajapati H.P. et al.** | **Razak A.A. et al.** | **Sarkar S. et al.** | **Savik R. et al.** | **Shimony N. et al.** | **Song S. et al.** | **Starke R.M. et al.** | **Trimpou P. et al.** | **Wang A.C. et al.** | **Zaidi H.A. et al.** | **Zhang C. et al.** | **Zhang T. et al.** |
| Clearly stated research question | Yes | Yes | Yes | Yes | Yes | Yes | Yes | Yes | Yes | Yes | Yes | Yes | Yes | Yes | Yes | Yes | Yes | Yes | Yes | Yes | Yes | Yes | Yes | Yes | Yes |
| Defined study population | Yes | Yes | Yes | Yes | Yes | Yes | Yes | Yes | Yes | Yes | Yes | Yes | Yes | Yes | Yes | Yes | Yes | Yes | Yes | Yes | Yes | Yes | Yes | Yes | Yes |
| ≥50% participation rate | NR | Yes | Yes | Yes | Yes | Yes | NR | NR | Yes | Yes | NR | Yes | Yes | NR | Yes | Yes | NR | Yes | Yes | Yes | Yes | Yes | Yes | Yes | NR |
| Uniform inclusion/exclusion criteria | NR | Yes | Yes | NR | Yes | Yes | Yes | NR | Yes | NR | Yes | Yes | Yes | Yes | NR | Yes | Yes | Yes | Yes | Yes | Yes | Yes | Yes | Yes | NR |
| Justification of sample size | No | No | No | No | No | No | No | No | No | No | No | No | No | No | No | No | No | No | No | No | No | No | Yes | No | No |
| Exposure assessed before outcome | Yes | Yes | Yes | Yes | Yes | Yes | Yes | Yes | Yes | Yes | Yes | Yes | Yes | Yes | Yes | Yes | Yes | Yes | Yes | Yes | Yes | Yes | Yes | Yes | Yes |
| Sufficient follow-up period | NR | NR | Yes | Yes | Yes | Yes | Yes | Yes | Yes | Yes | Yes | Yes | Yes | Yes | Yes | Yes | Yes | Yes | Yes | Yes | Yes | Yes | Yes | Yes | NR |
| Measurable/consistent exposure assessment | Yes | Yes | Yes | Yes | Yes | Yes | Yes | Yes | Yes | Yes | Yes | Yes | Yes | Yes | Yes | Yes | Yes | Yes | Yes | Yes | Yes | Yes | Yes | Yes | Yes |
| Clearly defined outcome measures | Yes | Yes | Yes | Yes | Yes | Yes | Yes | Yes | Yes | Yes | Yes | Yes | Yes | Yes | Yes | Yes | Yes | Yes | Yes | Yes | Yes | Yes | Yes | Yes | Yes |
| Blinded outcome assessment | No | No | Yes | No | No | No | No | No | No | No | No | No | No | No | Yes | No | No | Yes | No | No | No | Yes | No | No | No |
| ≤20% loss to follow-up | NR | NR | Yes | NR | NR | NR | NR | NR | Yes | NR | NR | Yes | NR | NR | Yes | NR | NR | Yes | Yes | Yes | Yes | NR | Yes | Yes | NR |
| Confounders measured and adjusted | Yes | Yes | Yes | Yes | Yes | Yes | Yes | Yes | Yes | Yes | Yes | Yes | Yes | Yes | Yes | Yes | Yes | Yes | Yes | Yes | Yes | Yes | Yes | Yes | Yes |
